# Potential application of peripheral blood biomarkers in intracranial aneurysms

**DOI:** 10.3389/fneur.2023.1273341

**Published:** 2023-10-19

**Authors:** Yangying Wu, Ziya Zhao, Shaolei Kang, Lijuan Zhang, Fajin Lv

**Affiliations:** ^1^Department of Radiology, The First Affiliated Hospital, Chongqing Medical University, Chongqing, China; ^2^The Department of Medical Imaging, The First Affiliated Hospital of Kunming Medical University, Kunming, China

**Keywords:** blood biomarker, intracranial aneurysm, inflammation, cytokine, blood lipid

## Abstract

Intracranial aneurysm (IA) counts are increasing yearly, with a high mortality and disability after rupture. Current diagnosis and treatment rely on costly equipment, lacking effective indicators for progression prediction and specific drugs for treatment. Recently, peripheral blood biomarkers, as common clinical test samples, reflecting the immune and inflammatory state of the body in real-time, have shown promise in providing additional information for risk stratification and treatment in IA patients, which may improve their outcomes after aneurysm rupture through anti-inflammatory therapy. Therefore, this paper reviewed the progress of potential biomarkers of IAs, including inflammatory blood indicators, cytokines, and blood lipids, aiming to aid individual management and therapy of aneurysms in clinical practices.

## 1. Introduction

Intracranial aneurysms (IAs) are common public health events and have similarities with atherosclerosis in histology, characterized by inflammation and vascular wall structure degeneration (1, 2). The occurrence rate of unruptured IAs (UIAs) is about 3.2% in patients worldwide without comorbidity and at an average age of 50 (3). Aneurysms have an annual rupture rate of 1.4% and a 5-year rupture risk of 3.4% (4), but subarachnoid hemorrhage (SAH) caused by IAs rupture has a high mortality and disability (5). IAs are traditionally treated with surgical clamping or intravascular intervention. Even if patients are treated timely, they presumably experience complications such as hydrocephalus, cerebral vasospasm, and neurological dysfunction, which burden their families and society. Detecting UIA is essential, but current approaches such as digital subtraction angiography, computed tomography angiography, and magnetic resonance angiography are expensive, with strict indications, contraindications, potential contrast, and radiation load. Besides, patients with UIA remain temporarily stable, and long-term follow-ups are performed under traditional imaging technologies. However, follow-up time varies from person to person and is also difficult to determine. Finding practical, efficient, and economical methods to distinguish rupture-prone IAs is necessary.

Blood testing is a routine clinical examination with the advantages of convenience, noninvasiveness, low economic cost, and a broad application scope that provides timely information on the immune function status of the body. Researchers and clinical doctors have focused on the correlation between peripheral blood biomarkers and IAs, which offers important insights into early risk evaluation and treatment of IAs. Whether combining the expression levels of these indicators in the blood is effective for IAs assessment remains unclear. This article summarizes the progress of related research to provide a reference for the personalized management and treatment of IAs.

## 2. Inflammation and intracranial aneurysms

IAs are pathological protrusion or dilation of intracranial vascular walls caused by congenital or acquired factors with complex pathophysiological mechanisms involving genetics, environment, and epidemiology. Extensive research (6–9) demonstrated that hemodynamics, inflammation, atherosclerosis, and hypercholesterolemia might all pertain to the formation and rupture of IAs. Vascular endothelial cell dysfunction caused by hemodynamics led to an inflammatory response involving various cytokines, inflammatory mediators, macrophages, T cells, NK cells, and neutrophils. Subsequently, smooth muscle cells underwent pro-inflammatory phenotype regulation; inflammatory responses contributed to the destruction of the internal elastic layer and the digestion of the extracellular matrix in the vascular wall, eventually forming an aneurysm. The loss of wall cells, further inflammation, and vascular wall degeneration ultimately resulted in IAs rupture (10). In a histological and hemodynamic study, Liu et al. (11) reported a correlation between inflammation and biomechanical stress on aneurysm walls.

Recently, there has been a growing interest in the influence of oral and gastrointestinal microbiota on IAs. Li et al. (12) conducted a case–control metagenome-wide association study, which revealed that changes in gut microbiota were associated with the progression of UIAs. In an animal model, Shikata et al. (13) observed macrophage infiltration and mRNA levels of inflammatory cytokines changed with gut microbiota depletion, suggesting that gut microbiota played a critical role in the pathophysiology processes of forming an aneurysm by regulating inflammation. Moreover, Hallikainen et al. (14) reported that periodontitis and gingival bleeding also correlated with the higher formation and rupture risk of IAs. Based on the hypothesis of an association between oral infections and inflammation of the IA wall, Hallikainen et al. (15) deeply explored several potential mechanisms by which periodontitis and periodontal pathogens might increase the risk of aneurysm rupture by promoting degenerative remodeling of the aneurysm wall. These provided a new insight for us to understand the pathogenesis of IAs better. However, the specific role of bacterial biomarkers in IAs still needs further investigation.

## 3. Application of peripheral blood biomarkers in unruptured intracranial aneurysms

UIAs with significant clinical symptoms or evidence of growth during follow-up were generally associated with a higher risk of rupture, indicating disease progression (16, 17). Besides, vascular wall enhancement was positively correlated with the rupture risk of IAs, while the absence of wall enhancement suggested that aneurysms were stable (18). Routine peripheral blood biomarkers such as white blood cells, cytokines, and blood lipids are economical and clinically accessible, reflecting real-time functional activity situations of the body. Studies have found relevancy between these biomarkers and IAs progression, but the specific correlation degree should be evaluated comprehensively based on different indicators.

### 3.1. Cytokines and intracranial aneurysms

Cytokines are common biomarkers that mediate and regulate inflammation, immune response, cell proliferation, and other biological processes. Their significance in the field of IAs has gained attention. Kamińska et al. (19, 20) demonstrated that interleukin-8 (IL-8) and IL-6 concentrations in cerebrospinal fluid (CSF) were higher than in serum for patients with UIA. IL-8 concentration in CSF was significantly related to aneurysm size. Furthermore, IL-6 in CSF was higher in patients with multiple aneurysms than those with one. Besides, the IL-8 Quotient (ratio of IL-8 in CSF versus serum) and IL-6 Quotient (ratio of IL-6 in CSF to serum) both showed a potential role in the pathogenesis of aneurysm formation.

Liu et al. (21) prospectively investigated the correlation between imaging, cytokines, and histological samples of patients with UIA. They demonstrated that aneurysm wall enhancement was associated with pyroptosis in UIA tissues and pyroptosis-related serum cytokines such as the inflammatory cytokine IL-1β and anti-inflammatory cytokine IL-1 receptor antagonist (IL-1.ra). The IL-1.ra to IL-1β ratio in serum showed the most extraordinary ability in predicting aneurysm wall enhancement, with an area under the curve of 0.96, followed by IL-1.ra, IL-1β and tumor necrosis factor-alpha (TNF-α). They also found that aneurysm wall enhancement of UIAs showed a higher prevalence of vascular wall remodeling, along with increased levels of pyroptosis-related proteins (CD68 and MMP2), more severe inflammation, and larger areas of arterial atherosclerosis in tissues when compared to those without enhancement. The pathological characteristics were similar to those observed in ruptured aneurysms. Liu et al. (22) analyzed inflammatory markers in serum and histology of IAs based on multiple contrast groups, strictly controlling for traditional risks and confounding factors. They uncovered a correlation between IL-1 levels in serum and tissue, suggesting its potential as a biomarker for evaluating the instability of aneurysms, including ruptured or symptomatic cases. They found that the IL-1.ra to IL-1β ratio was highly accurate in assessing aneurysm rupture (C statistic =0.91) and provided a better discriminability for symptomatic aneurysms when combined with the PHASES score than using the PHASES score alone.

Yang et al. (23) prospectively collected preoperative blood samples from 184 UIA patients and divided them into high and low-risk groups based on propensity matching score and ELAPSS score; results showed patients with higher serum levels of IL-15, monocyte chemotactic protein-1 (MCP-1), and TNF-β were associated with an increased risk of aneurysm growth, and elevated IL-15 and TNF-β serum levels might indicate IA progression. Kamińska et al. (24) analyzed 27 cytokines in the serum and CSF of UIA patients and compared them to those without IA. Results showed that cytokines in CSF might be locally synthesized within the central nervous system, reflecting inflammatory response at the site of aneurysms and more effective than serum in identifying IA formation and development. Besides, they observed that both pro- and anti-inflammatory mechanisms activated in UIA patients, where IL-2, IL-6, IL-8, macrophage inflammatory protein-1 alpha, and MCP-1 were identified as the primary factors contributing to IA formation. Moreover, IL-1β, IL-6, IL-8, MCP-1, and TNF-α in CSF might represent potential preventive therapeutic targets due to their positive correlation with the size and number of IAs. Lately, Liu et al. (25) performed a large multicenter prospective cohort study, revealing the potential value of biomarkers such as oleic acid, arachidonic acid, IL-1β, and TNF-α in identifying unstable aneurysms (rupture or growth). Additionally, they established and validated a risk stratification model based on radiological features and biomarkers to aid treatment decision-making for patients with UIAs.

### 3.2. Blood inflammatory indicators and intracranial aneurysms

Kim et al. (26) analyzed peripheral blood cells from 1,209 patients with IA and suggested that platelet-to-neutrophil ratio and platelet-to-white blood cell ratio were significantly relevant to the risk of aneurysm rupture, higher neutrophil-to-lymphocyte ratio, and erythrocyte sedimentation rate, lower platelet-to-neutrophil ratio and platelet-to-white blood cell ratio indicated a higher PHASES score in individuals with UIA. Zhang et al. (27) collected clinical data from 937 patients with 1,088 UIAs and found a positive dose–response relationship between neutrophil-to-lymphocyte ratio levels and the stability scores of aneurysms, including PHASES score, ELAPSS score, and JAPAN 3-year rupture risk score. They also noted that the higher the neutrophil-to-lymphocyte ratio, the greater the risk of aneurysm growth.

The systemic immune inflammation index, a novel immune inflammatory marker, integrates platelet, neutrophil, and lymphocyte counts in peripheral blood, effectively reflecting inflammation levels and immune status in the whole body. It was first developed by Hu et al. (28) in the prognosis study of hepatocellular carcinoma, subsequently widely used in lung cancer (29, 30), gastric cancer (31), and other disease prognosis research. Lately, Peng et al. (32) confirmed that the systemic immune inflammation index was an independent predictor for fusiform IAs with aneurysm wall enhancement, with higher values indicating a higher occurrence of aneurysm wall enhancement; the study also found that levels of neutrophils, neutrophil-to-lymphocyte ratio, and the systemic immune inflammation index were higher in patients with aneurysm-related symptoms than in asymptomatic aneurysms.

### 3.3. Blood lipids and intracranial aneurysms

The role of peripheral blood lipids in IAs is a highly studied area. Tian et al. (33) performed a Mendelian randomization study and found a significant causal relationship between obesity and IAs. There was a positive correlation between the fat percentage in the body and the risk of IA rupture. In animal experiments, Shimizu et al. (34) confirmed that elevated serum cholesterol levels promoted the loss of blood vessel medial smooth muscle layers and the enlargement of IAs through foam cell transformation. Ou et al. (35) used a patient-specific simulation of low-density lipoprotein transport mode and confirmed that increased lipid accumulation might exacerbate IAs rupture. They also found that higher lipid infiltration tended to occur at the rupture-prone site of the aneurysm, such as the tip or bleb. Zhong et al. (36) illustrated that atherosclerosis was the sole predictor of aneurysm wall enhancement, characterized by inflammatory cell infiltration within atherosclerosis, intraluminal thrombus, and vasa vasorum.

## 4. Application of peripheral blood biomarkers in ruptured intracranial aneurysms

SAH is mainly due to the rupture of IAs and may accompany potential complications such as cerebral vasospasm, delayed cerebral ischemia (DCI), rebleeding, etc. Timely diagnosis, treatment, and meticulous management are crucial for reducing the risk of complications and improving prognosis.

### 4.1. Cytokines and intracranial aneurysms

Values of cytokines in evaluating prognosis for aneurysmal SAH (aSAH) have sparked considerable attention. Elevated peripheral C-reactive protein (CRP) and IL-6 levels existed in aSAH patients with poor prognoses (37). According to Schranz et al. (38), tumor necrosis factor superfamily 14 and oncostatin-M serum levels were higher in aSAH patients within 24 h after symptoms onset than healthy controls. Lower tumor necrosis factor superfamily 14 was associated with the incidence of 30-day mortality and DCI. Furthermore, elevated tumor necrosis factor superfamily 14 levels were identified as an independent predictor of survival. Interestingly, the concentration of oncostatin-M showed a positive relationship with tumor necrosis factor superfamily 14 in survivors. Subsequently, Schranz et al. (39) proposed that serum levels of fatty acid-binding protein 3 and CXC-chemokine ligand 16 tested at 24 h after symptom onset were significantly correlated with a 30-day unfavorable outcome (modified Rankin score 3–6) in patients with aSAH, but not associated with DCI. In addition, the study found that CXC-chemokine ligand 16 (>446.7 pg./mL) could independently predict poor outcomes.

Chen et al. (40) reported that serum macrophage migration inhibitory factor could independently predict unfavorable outcomes 6 months after aSAH. Later, Yang et al. (41) showed that higher serum levels of macrophage migration inhibitory factor independently predicted DCI following aSAH and had superior prognostic value compared to the Acute Physiology and Chronic Health Evaluation II score, CRP, IL-6, and the Hunt and Hess grade. Besides, combining macrophage migration inhibitory factor with IL-6 and CRP enhanced the predictive efficacy of macrophage migration inhibitory factor in assessing DCI. Neumaier et al. (42) first investigated the changes in macrophage migration inhibitory factor levels in serum, global cerebral, and local cerebral during the first 3 weeks after aSAH. Their findings suggested that aneurysms in the anterior circulation might associated with higher levels of macrophage migration inhibitory factor in serum. Additionally, increased levels of macrophage migration inhibitory factor in serum and cerebral microdialysate during Days 5–15 were potentially related to DCI following aSAH.

Spantler et al. (43) analyzed a multiplex serum biomarker in 112 patients with aSAH at two time points. Their findings illustrated that monocyte chemotactic protein-3 and chemokine (C-X3-C motif) ligand-1 concentrations at Day 5–7 were relevant to DCI after aSAH. Additionally, on Day 1, higher levels of interferon gamma-induced protein 10, monocyte chemotactic protein-3, and macrophage inflammatory protein-1 beta were observed in aSAH patients who had unfavorable outcomes on Day 30 (modified Rankin scale ≥3) compared to those with favorable outcomes. Moreover, elevated IL-4 levels detected on Day 5–7 were found in patients with Transcranial Doppler spasm. This research emphasized the significance of analyzing the long-term kinetics of these biomarkers.

In another study, Csecsei et al. (44) examined the kinetics of serum ADAMTS13, growth differentiation factor-15, and neutrophil gelatinase-associated lipocalin during the initial stage of aSAH. They demonstrated that growth differentiation factor-15 was a reliable predictor of poor functional outcomes at 3 months among aSAH patients, even those with low-grade status (World Federation of Neurosurgical Societies score grade I-III) at admission. Neutrophil gelatinase-associated lipocalin in the early stage showed a significant correlation with macrovascular vasospasm following SAH; ADAMTS13 detected on days 7 and 9 was also associated with this condition.

### 4.2. Blood inflammatory indicators and intracranial aneurysms

Neutrophils are the most abundant white blood cells, functioning as the first line of defense against acute infections or inflammation in the immune system. Cuoco et al. (45) analyzed immune cell counts in 143 patients with aSAH, implying that those with higher neutrophil counts (≥9.80 × 10^3^/mL) upon admission were more likely to develop acute symptomatic hydrocephalus and dependent shunting. Zhang et al. (46) conducted a large-scale, multicenter propensity score matching study, analyzing blood inflammatory factors of 6,041 patients with aSAH on admission; results showed that patients with higher neutrophil counts had higher in-hospital mortality, hospital-acquired infection rates, and delayed neurological ischemic deficits.

CRP is a non-specific marker of inflammation and tissue damage. Median CRP levels were higher in patients with fusiform IA than those with saccular IA and not obviously influenced by diabetes, smoking status, hypertension, and sex. CRP also could independently forecast the morphology of fusiform UIAs (47). Moreover, Yang et al. (48) illustrated that high-sensitivity CRP levels over 6.6 mg/L could predict acute kidney injury after aSAH with a sensitivity of 76.5% and a specificity of 64.6%. Besides, Ho et al. (49) reported that the high-sensitivity CRP was potentially valuable in predicting cerebral vasospasm and systemic infection in patients after aSAH. According to Rasmussen et al. (50), high plasma levels of high-sensitivity CRP significantly correlated with both vasospasm and clinical outcomes after aSAH.

Apart from individual inflammatory markers, comprehensive immune parameters may detect more subtle changes in peripheral blood associated with the prognoses of aSAH, which involve more extensive information and may compensate for the limitations of using a single blood indicator. In a retrospective cohort study spanning 10 years, Zhang et al. (51) highlighted the importance of inflammatory indicators in predicting aSAH prognosis. Their results demonstrated that incorporating the neutrophil-to-albumin ratio into existing prediction models, such as SAFIRE (52) and SAHIT (53), significantly improved the ability to predict the 3-month mortality of aSAH patients. Moreover, a higher neutrophil-to-lymphocyte ratio level reflected a heavier systemic inflammation, indicating a higher risk of poor outcomes in patients with aSAH, more likely to result in rebleeding, DCI, inpatient death, pneumonia during hospitalization, and other unfavorable functional outcomes (54–58).

The systemic inflammatory response index was a novel composite parameter widely used in IA research; first proposed by Qi et al. (59) in a pancreatic cancer study, defined as follows: the systemic inflammatory response index = N × M/L, the N, M, and L was the peripheral neutrophil, monocyte, and lymphocyte counts before treatment. Zhang et al. (60) stated that the systemic inflammatory response index was an independent risk factor for unfavorable outcomes in aSAH patients. Nie et al. (61) retrospectively analyzed preoperative inflammatory biomarkers in 543 patients with aSAH; results identified inflammatory biomarkers, including white blood cell count, the systemic inflammatory response index, neutrophil count, neutrophil-to-albumin ratio, monocyte count, and monocyte-to-lymphocyte ratio were independently correlated with patients’ 90-day adverse outcomes, where white blood cells demonstrated the most incredible predictive accuracy. Yun et al. (62) indicated that an increase in the systemic inflammatory response index and systemic immune inflammation index marked a higher risk of poor outcomes in patients with aSAH. Furthermore, Geraghty et al. (63) demonstrated that the systemic immune inflammation index could also be an independent predictor of delayed cerebral vasospasm in patients with aSAH, with higher values indicating a higher risk.

It should be mentioned that acute inflammation after aSAH was associated with adverse functional outcomes. However, atherosclerosis, widely considered a chronic inflammation process, was present in all IAs, and its progression within IA positively related to IA growth (64, 65). In this regard, targeted therapy holds potential application value in reducing inflammation. By dynamically monitoring relevant signaling molecules, it aided in evaluating the systemic inflammatory levels and the efficacy of targeted treatment. This approach also facilitated a better understanding of alterations in peripheral blood cell levels associated with an acute infection due to post-SAH immunodepression or chronic low-grade inflammation related to atherosclerosis (66, 67). Therefore, investigating the dynamic changes in peripheral blood inflammatory biomarkers is critical for both risk and prognosis evaluation of IAs.

## 5. Potential drug therapy approaches in intracranial aneurysms

Statins, widely used lipid-regulating drugs in clinical practice, play a vital role in preventing and treating cardiovascular diseases. Using statin for IAs has gradually emerged as a popular research topic in recent years, but viewpoints are inconsistent. Some believed that statin played a potential protective role in aneurysm rupture prevention. Peng et al. (68) found that apolipoprotein B and statin use were independently related to aneurysm wall enhancement of fusiform UIAs, indicating statin might suppress the inflammatory processes of the aneurysmal wall to decrease the risk of aneurysm growth and rupture. Besides, total cholesterol, low-density lipoprotein, and apolipoprotein B seemed to positively related to the maximal diameter of fusiform IAs. Kang et al. (69) conducted a prospective randomized controlled trial and suggested that statin could reduce the aneurysm wall enhancement of UIAs, and it showed a potential ability to alleviate inflammation in the aneurysm wall.

However, Wang et al. (70) demonstrated that atorvastatin lowered the risk of aneurysm growth but was unrelated to aneurysm rupture in a large-sample, multicenter prospective study. In addition, a meta-analysis (71) also mentioned that statin therapy did not trigger IA rupture; individuals with hyperlipidemia experienced a reduced risk of rupture. Nevertheless, the preventive effect of statins on the growth of small UIAs not demonstrated in an open-label, multicenter, randomized controlled trial conducted by Yoshida et al. (72). Moreover, the management guidelines issued by the European Stroke Organization directed that current data did not suggest any beneficial or detrimental effect for statins in the prevention of aneurysm rupture or growth (73). Naraoka et al. (74) illustrated that long-acting statins significantly reduced the occurrence of cerebral vasospasm after aSAH; however, they did not improve patients’ outcomes. One multicenter retrospective study recently also proved statins could not improve clinical outcomes of IA patients who underwent pipeline embolization device treatment (75). Overall, the efficacy of statins in preventing and treating IAs remains unclear. Further large-scale prospective studies are still needed.

Expect statins, researchers are also interested in exploring new anti-inflammatory medications for aSAH therapy, such as vitamin D. Guan et al. (76) first reported a notably elevated incidence of hypovitaminosis D among patients who required IAs treatment. Wei et al. (77) proposed that vitamin D levels were related to IAs rupture and were lower in aSAH patients than those with non-aneurysmal SAH. Kashefiolasl et al. (78) concluded that vitamin D reduced cerebral vasospasm following SAH by inducing stroma-cell-derived factor 1α, suggesting its potential as a treatment agent for cerebral vasospasm. However, Alvarado Reyes et al. (79) observed that vitamin D deficiency was unrelated to the outcomes of patients with aSAH.

Colchicine is an anti-inflammatory medication characterized by a favorable safety profile and available at a low cost. A meta-analysis (80) suggested that using colchicine might reduce the risk of stroke in patients under high cardiovascular risk. It showed success in preventing atherosclerotic cerebrovascular disease. Zhao et al. (81) conducted an animal study that demonstrated the protective effect of colchicine against the development of experimental abdominal aortic aneurysms. However, Phie et al. (82) suggested that colchicine lacked efficacy in reducing the growth of abdominal aortic aneurysms in a mouse model. Although these findings primarily pertained to abdominal aortic aneurysms, they inspired intriguing possibilities regarding the potential applications of colchicine in managing IAs. Further research is warranted to unravel the precise mechanisms of action and evaluate the efficacy of colchicine, specifically in the context of IAs.

In addition to exploring pharmacological interventions, researchers have also focused on addressing the complications that can arise from aSAH. Paroxysmal sympathetic hyperactivity was considered a potential complication of aSAH, which was life-threatening and associated with severe brain injury but lacked specific clinical symptoms (83). Mathew et al. (84) performed a prospective study that demonstrated a correlation between paroxysmal sympathetic hyperactivity after severe traumatic brain injury and more extended hospital stay, worse Disability Rating Scale at discharge, higher mortality, and unfavorable outcomes (including death, vegetative state, or severe disability). They used clonidine, an α2 receptor agonist, and supportive therapy to alleviate relative symptoms. However, they did not compare to those without clonidine treatment. Clonidine was considered helpful in treating paroxysmal sympathetic hyperactivity (85). Another study (86) proposed that combining adrenergic blockade with propranolol and clonidine in patients with severe traumatic brain injury was safe and feasible, but it did not significantly alter patients’ outcomes, except for improving features associated with sympathetic hyperactivity.

## 6. Limitations and prospects

Conventional peripheral blood biomarkers have potential clinical values in the early risk classification of UIAs and prognosis prediction of aSAH. They may facilitate less invasive and cost-effective exploration of IAs pathophysiology, thereby supporting the management of UIAs through preventative approaches. They also hold a promise to improve accuracy in assessing IA progression risk and expediting patients’ recovery. However, challenges exist in applications: peripheral blood inflammatory markers reflect systemic immune status rather than the local inflammation of IAs; they may be influenced by comorbidities, medication history, individual differences, and standardization of blood sample processing procedures. Moreover, single indicators have relatively limited accuracy, sensitivity, and specificity for predicting rupture risk and prognosis. A comprehensive analysis combining clinical manifestations with imaging findings and other biochemical markers like immune cells, cytokines, and blood lipids will emerge as a new trend in future IAs research. Standardized biological sample collection and processing protocols are required to reduce heterogeneity and make blood test results more accurate and reliable. Exploring the inflammation-associated signaling pathway in IAs and developing composite blood indices while dynamically detecting biomarkers will enhance their clinical application potential, ultimately achieving personalized management for IA patients.

## 7. Conclusion

Cytokines and peripheral blood inflammatory markers, particularly composite indices, highlighted significant values in predicting patients’ prognosis after aSAH and exhibited potential relevance to IAs progression. Besides, blood lipids might be associated with the risk rupture of IAs. Furthermore, anti-inflammation drugs may function as novel therapeutic interventions for IAs after further verification in clinical practices. There is no definitive single candidate blood biomarker used for assessing rupture risk or predicting prognosis until now. Therefore, there is an urgent need to develop new comprehensive blood indicators to optimize the management of IA patients. Future large-scale prospective research based on robust, standardized data should be enhanced to deepen our understanding of this complex disease and facilitate innovative treatment for IAs.

## Author contributions

YW: Investigation, Writing – original draft, Writing – review & editing. ZZ: Investigation, Writing – review & editing. SK: Writing – review & editing. LZ: Writing – review & editing. FL: Writing – review & editing, Supervision.
